# Mechanical Design and Assessment of a Low-Cost 7-DOF Prosthetic Arm for Shoulder Disarticulation

**DOI:** 10.1155/2018/4357602

**Published:** 2018-09-03

**Authors:** José-Alfredo Leal-Naranjo, Christopher-René Torres-San Miguel, Marco Ceccarelli, Horacio Rostro-Gonzalez

**Affiliations:** ^1^Departamento de Electrónica, DICIS, Universidad de Guanajuato, 36885 Salamanca, GTO, Mexico; ^2^Instituto Politécnico Nacional, Sección de Estudios de Posgrado e Investigación, Unidad Profesional “Adolfo López Mateos”, Escuela Superior de Ingeniería Mecánica y Eléctrica Zacatenco, Edificio 5, 2° Piso Col. Lindavista, 07738 México City, Mexico; ^3^Laboratory of Robotics and Mechatronics (LARM), DiCEM, University of Cassino and Southern Lazio, 03043 Cassino, Italy

## Abstract

This work presents the design of a low-cost prosthetic device for shoulder disarticulation. A proper design of the mechanisms has been addressed to obtain a prototype that presents 7 degrees of freedom. Shoulder movement is achieved by means of a spherical parallel manipulator, elbow movement is performed by a six-bar mechanism, and the wrist movement is implemented by a spherical parallel manipulator. A set of dynamic simulations was performed in order to assess the functionality of the design. The prototype was built using 3D printing techniques and implementing low-cost actuators. An experimental evaluation was carried out to characterize this device. The result of this work is a prototype that weighs 1350 g that is able to perform movements related to activities of daily living.

## 1. Introduction

One of the main issues related to the design of prosthetic devices is to mimic as close as possible the human motion. It has been demonstrated that the reduced dexterity of prosthetic arms yields to compensatory movements that could cause injuries in the long-term use [1]. Furthermore, the low functionality of the device causes overuse syndrome in people with upper limb deficiency [2]. Besides, the more proximal the amputation, the higher is the rate of abandonment of prosthetic arms [3]; this is mainly due to a low functionality and comfort. Shoulder disarticulation shows the lowest rate of incidence, so there is low incentive to develop solutions for people with this disability, and therefore, few prosthetic devices have been designed [4]. The amputation of the shoulder involves the necessity of more DOFs in the prosthetic device, and consequently, the complexity of the system increases.

Currently, some commercial solutions exist for different levels of arm amputation; among these, the most advanced prosthetic upper limbs available in the market are the *i-limb Ultra*, the *bebionic® v2 hand*, the *Contineo Multi-Grasp®*, and the *Michelangelo®* [5]. The patients with above-elbow amputation and shoulder disarticulation are the most difficult cases to approach; this is because more functional segments are needed in the prosthetic device and the difficulty of its design increases.

To the best of our knowledge, very few research works have been developed related to the design of a total upper limb. A design sponsored by the Defense Advanced Research Projects Agency (DARPA) of the USA is presented in [6]. This is a 4.8 kg device with different configurations and consists of a two-DOF actuated shoulder, a humeral rotator, an elbow, and a battery, which has been characterized by 26 DOFs (including the hand). The complexity of this device makes it unaffordable for most people.

The DEKA arm is one of the most advanced upper limb prostheses [7]. Patients with different amputation levels can use this device. It is the result of a project sponsored by DARPA. It shows a modular configuration that can be adapted to different amputation levels. The configuration for shoulder disarticulation has 10 DOFs that are distributed with six DOFs in the arm and four in the hand with a weight of 4.5 kg. This prosthesis can be controlled by different signals like switches and myoelectric signals and even with targeted reinnervation [8].

A 7-DOF prosthetic upper limb is presented in [9]. This prototype includes an underactuated hand with 15 DOFs. This device is based on differential mechanisms, where the load is shared between two motors, allowing the use of smaller actuators. The total weight of this system is 4.45 kg, which makes it heavy for use for a long time.

A survey on the current prosthetic arms shows that these solutions have been generally designed with fewer DOFs than the required ones. These designs present the limitations of low functionality and high weight. Furthermore, the most sophisticated prosthetic arms are unaffordable for most of the population and have been designed by long-term projects sponsored by the US Army.

Considering the previously outlined, in this work, we propose a light and easy-to-afford upper limb prosthesis for people with shoulder disarticulation that allows mimicking the movement of an arm through a set of prescribed trajectories.

## 2. Design of a Prosthetic Arm

The open issues referring to the upper limb prosthesis are the lack of functionality and discomfort due to high weight and that most of the devices are unaffordable.

The necessity of more functional segments presented in devices for shoulder disarticulation makes the mechanical design of the prosthetic arm an important topic. The prototype developed in this work has seven degrees of freedom achieved by a 3-DOF shoulder, a 1-DOF elbow, and a 3-DOF wrist. A preliminary design where the feasibility of the mechanism is shown is presented in [10].

## 3. Shoulder Design

The available space to allocate the shoulder mechanism makes the design of the shoulder a challenging task if an anthropomorphic shape is required. The prosthetic shoulder supports the entire structure of the device; therefore, the largest joint loads are developed in the shoulder.

**Figure 1:**
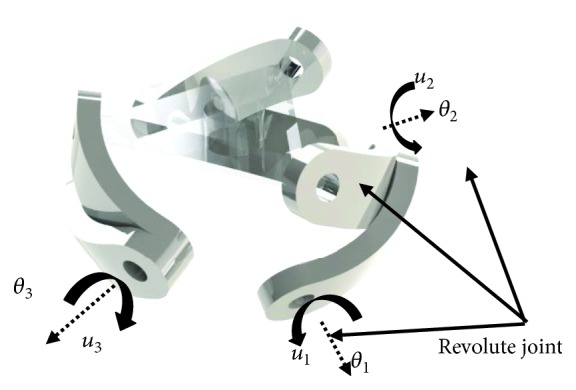
3-RRR parallel manipulator.

The shoulder mechanism is modeled as a 3-RRR-type spherical parallel manipulator (Figure 1). The dimensional synthesis of this manipulator was carried out using a multiobjective optimization based on genetic algorithms [11].

After the dimensional synthesis, from the inverse kinematics of the manipulator, it was observed that the links of the manipulator only perform rotations of an amplitude smaller than 90°. Considering this, it was proposed to include a mechanism in order to increase the torque that the motors exert to the links of the spherical manipulator.

**Figure 2:**
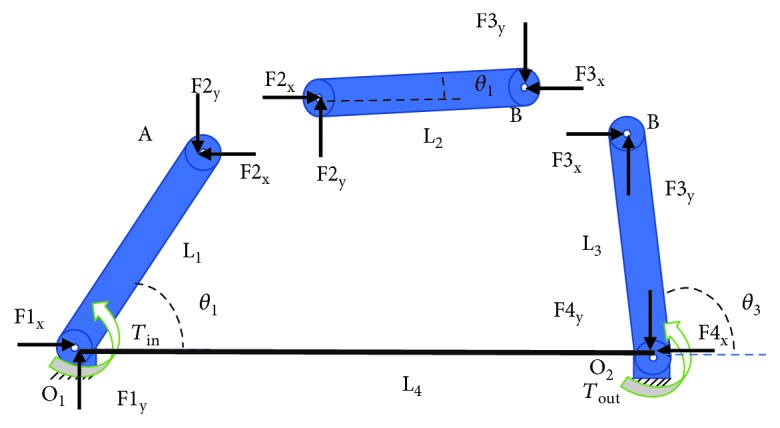
Free body diagram of the four-bar mechanism that joins the actuator and the base link of the parallel manipulator.

The proposed mechanism to achieve a mechanical advantage is a four-bar mechanism that is installed before the designed solution. A single-objective optimization procedure was carried out to define the link lengths. The objective function was the minimization of the maximum torque exerted by the motor. A static analysis of the four-bar mechanism was performed as shown in Figure 2.

From the free body diagram in Figure 2, an equation can be found to evaluate the torque of the motor (*τ*_in_) as a function of the parameters of the mechanism and the torque that requires the manipulator (*τ*_out_) for a defined task. This can be established as

(1) $$\begin{array}{c} Ax=b, \end{array}$$

where **A** is the coefficient matrix that depends on the link lengths and the position of the mechanism, *x* = [F1_x_, F1_y_, F2_x_, F2_y_, F3_x_, F3_y_, F4_x_, F4_y_, *τ*_in_], and *b* = [0, 0, 0, 0, 0, 0, 0, 0, *τ*_out_].

**Figure 3:**
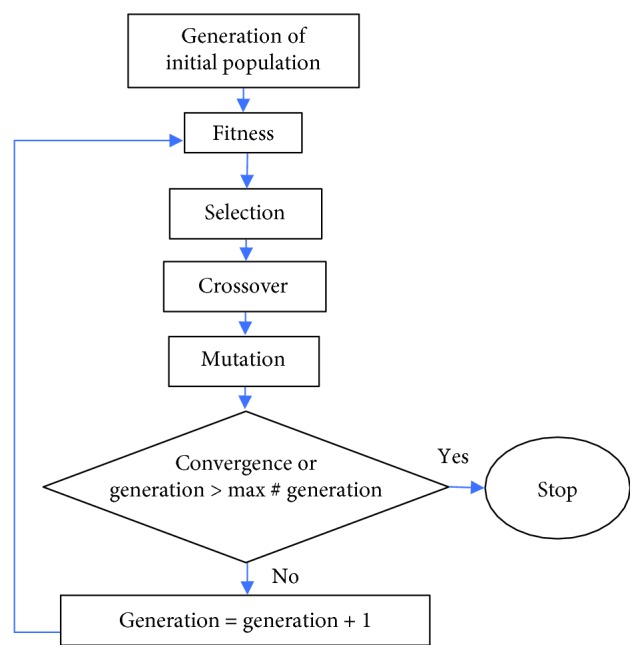
A flowchart of a genetic algorithm optimization.

From previous dynamic simulations [11], *τ*_out_ and *θ*_3_ are known values. An optimization based on GA was carried out using the algorithm in Figure 3. The chosen parameters were the link lengths (*L*_1_, *L*_2_, *L*_3_, and *L*_4_). An initial population of 200 elements was set in order to have a wide diversity of elements, and the maximum number of iterations was set to 100.

**Figure 4:**
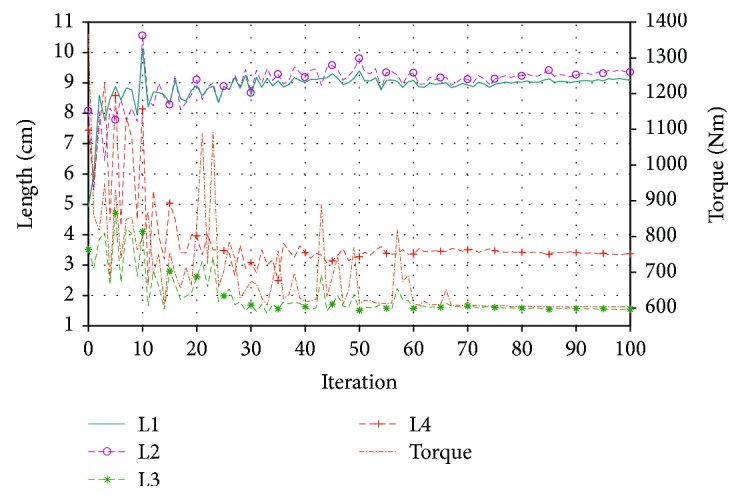
Evolution of the length links (left *y*-axis) and torque (right *y*-axis) during the optimization procedure.

**Figure 5:**
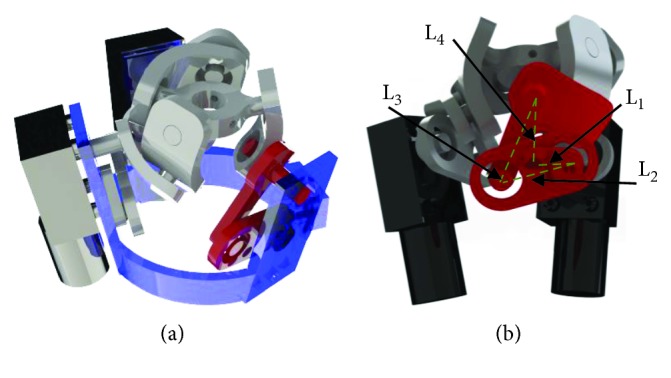
A CAD design of the final parallel manipulator with the four-bar mechanisms: (a) isometric view; (b) frontal view.

The initial population was created in a random way with link lengths ranging from 1 to 10 cm.  Figure 4 shows the evolution of the link lengths during the optimization process (left *y*-axis). It can be seen that convergence is reached approximately at iteration 70.  Figure 4 shows the evolution of the torque during the optimization process (right *y*-axis). This procedure was repeated for the three limbs of the parallel manipulator; thus, three different four-bar mechanisms were obtained. A four-bar mechanism attached to the parallel manipulator can be seen in Figure 5.

## 4. Elbow Design

**Figure 6:**
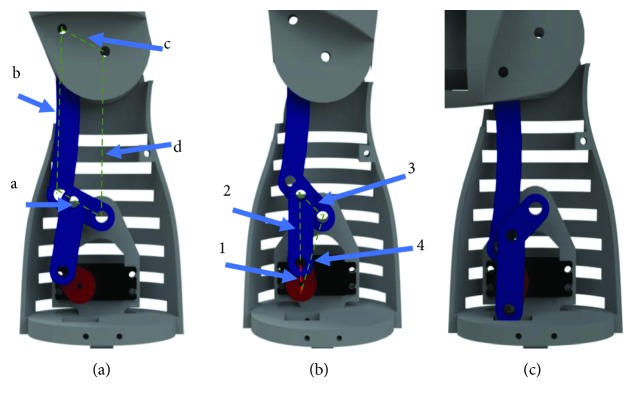
Mechanism design for the elbow flexion-extension: (a) the first four-bar mechanism; (b) the second four-bar mechanism in a singular position; (c) second singular position of the mechanism.

The flexion-extension of the elbow and the pronation-supination of the forearm can be modeled as two perpendicular revolute joints in series, but in this design, the pronation-supination is left as a movement of the wrist. The flexion-extension is performed using a six-bar mechanism that can be analyzed as two four-bar mechanisms. In the first part of the mechanism, links a and c have the same length as well as links b and d (Figure 6(a)). In this mechanism, the rotation of link c is the same as the rotation of link a. Link b is designed with a curvature in order to avoid collision with the external part of the prosthesis. The first four-bar mechanism is driven by a second one (Figure 6(b)). This mechanism is a singularity-based four-bar mechanism as suggested in [12]. As the input mechanism approaches a singularity, the output torque increases. The first link is attached to the actuator, and the third link is part of link a. The links of the second mechanism were designed in such a way that the range of motion of the third link is 90°; this is done to be able to complete the elbow flexion-extension motion. A graphical linkage synthesis was applied [13]. This design allows placing the actuator near to the shoulder, and in this way, the reduction of the moment of inertia is achieved.

## 5. Wrist Design

**Figure 7:**
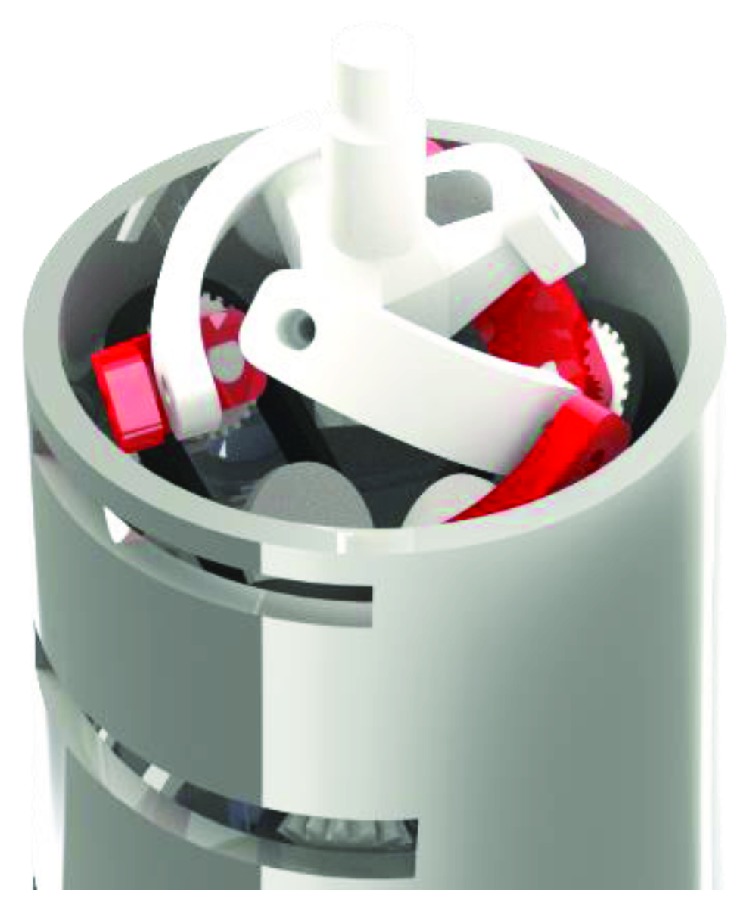
A CAD design of the parallel manipulator of the wrist.

The forearm pronation-supination, wrist flexion-extension, and wrist ulnar-radial deviation are achieved by means of a spherical manipulator that is placed at the wrist. This mechanism is used because the load is distributed into small motors that can be fitted inside the forearm structure (Figure 7).

To simplify, the design was considered a symmetrical architecture for the parallel mechanism. This means that the upper and lower pyramid of the manipulator are regular pyramids and are defined by the angles *γ* and *β* and were defined as suggested in [14]. As a symmetrical mechanism, the three legs are equal and there are only two parameters to define. Such parameters are the angles of the two links of each leg, *α*_1_ and *α*_2_. The dexterity and torque were calculated for different values of *α*_1_ and *α*_2_ ranging from 50° to 80, and it was concluded that the most suitable values for the size of the links were *α*_1_ = 60° and *α*_2_ = 80°.

**Figure 8:**
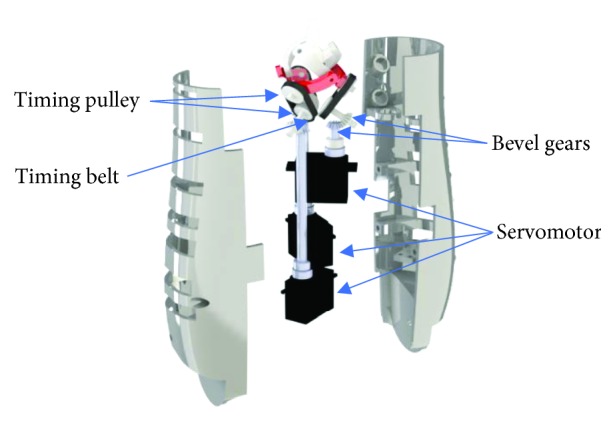
A CAD design of the transmission system of the wrist mechanism.

Three servomotors were placed inside the forearm. Each servo drives a bevel gear. There is a timing belt at the output side of the gear. A second timing belt is attached to the driving link of the parallel manipulator of the wrist (Figure 8).

According to [15], the required wrist range of motion (ROM) to perform different activities of daily living is 40° for wrist flexion and extension (each) and 40° of combined radial-ulnar deviation. This ROM is achieved with the selected parameters of the wrist mechanism.

## 6. Overall Design of the Prosthetic Arm

**Figure 9:**
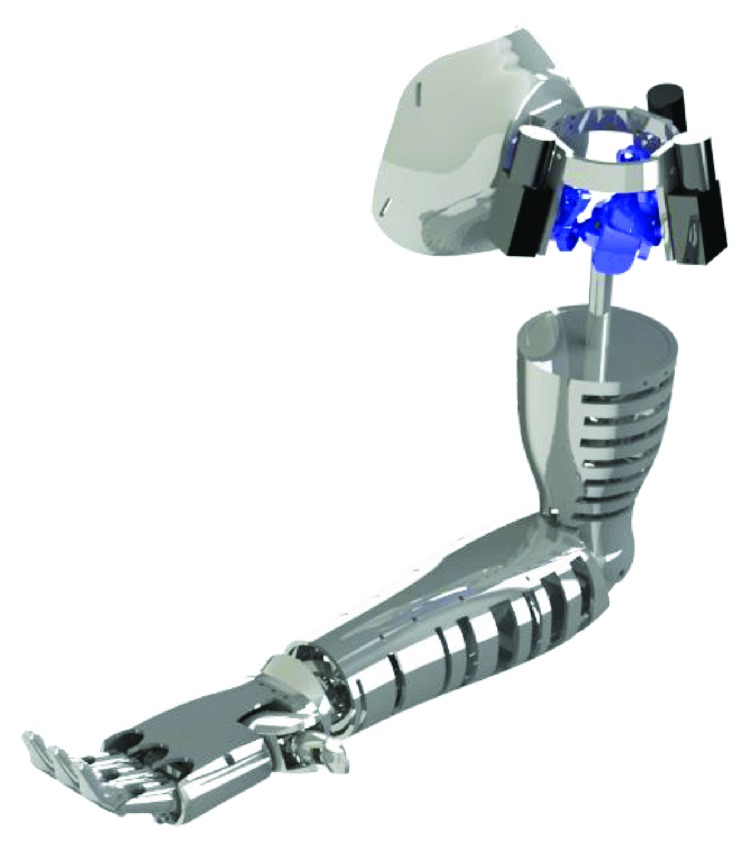
A CAD design of the prosthetic upper limb.

The proposed mechanisms were assembled together in a serial configuration to make a human-like upper limb prosthesis as shown in Figure 9, including a prosthetic hand design in a previous work [16]. The shoulder presents the three-DOF spherical manipulator with its actuators. The spherical mechanism allows the three movements of the shoulder using small motors. The end effector of the manipulator is attached to the forearm structure by means of a plate. The actuator that drives the elbow is placed at the proximal part of the forearm. With the described design, the prosthetic device can be constructed with a human-like size. Inside the prosthetic arm, there is enough space to place the batteries and control system.

## 7. Numerical Evaluation

Using the software MSC.ADAMS®, a set of dynamic simulations was performed in order to assess the relationship between the actuators and forces acting on the mechanism and the resulting acceleration, velocity, and motion trajectories. ABS with a density of 1020 kg/m^3^ was considered for the elements of the prosthetic arm. The weights of the actuators inside the arm were taken into account. At the joints of the shoulder mechanism of the prototype, deep groove ball bearings are located, so the friction coefficient was considered as 0.0015 [SKF, 2017]. In the remaining joints, the assembly was done using bolts, so the friction coefficient was considered as 0.1.

**Figure 10:**
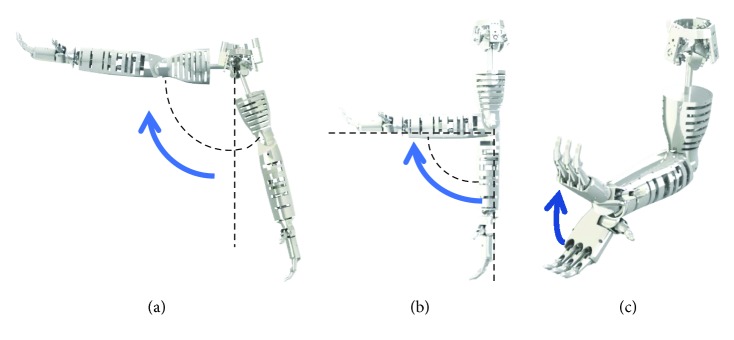
Simulation modes for the prototype: (a) humeral flexion with the elbow extended; (b) elbow flexion in a vertical position; (c) wrist flexion.

To evaluate the prototype, the simulations were defined as follows: Test I, a humeral flexion from −20° to 90° with the elbow extended and with a load of 5 N at hand (Figure 10(a)); Test II, an elbow flexion from 0° to 90° with the arm in vertical position and a load of 5 N at hand (Figure 10(b)); and Test III, a flexion from −40° to 40° of the wrist with the elbow flexed at 90° (Figure 10(c)).

Test I was performed in two different cases. In the first case, the experiment was carried out using the proposed four-bar mechanism attached to the parallel manipulator and with a duration of 2 s, and in the second case, it was performed actuating directly the parallel manipulator. Both cases were compared to show the performance of the implementation for the four-bar mechanism.

**Figure 11:**
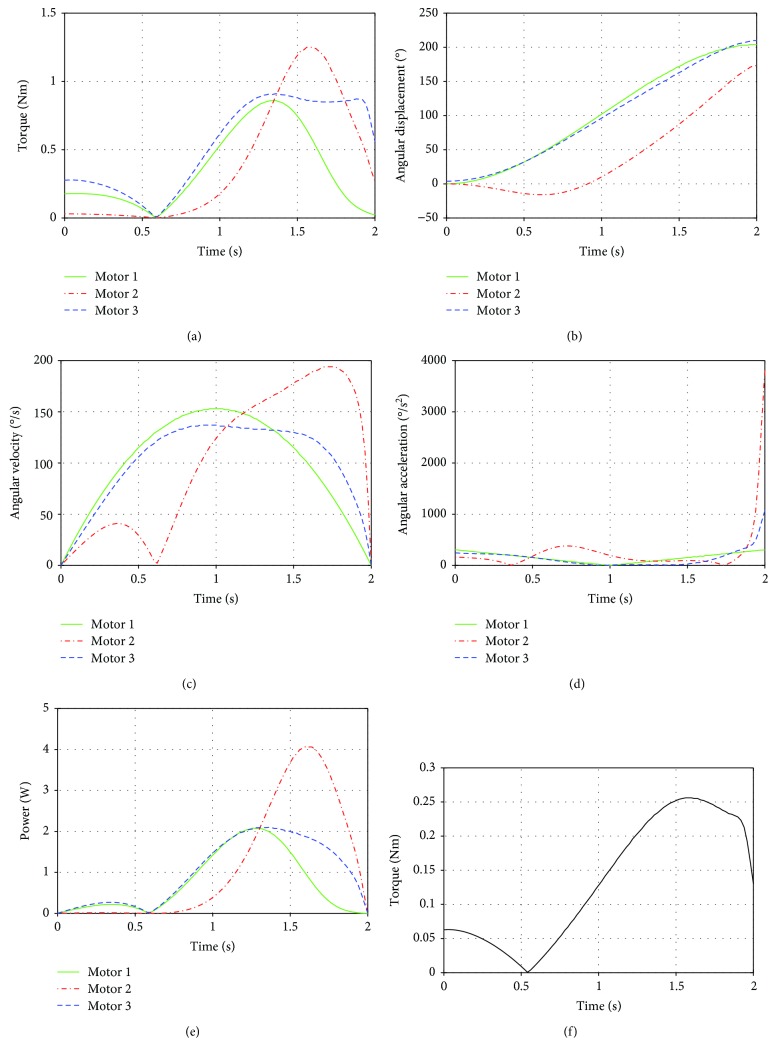
Results of Test I case a: (a) torques of the shoulder motors; (b) angular displacement; (c) angular velocity; (d) angular acceleration; (e) power of the motors of the shoulder; (f) torque of the elbow actuator.

For the first case in Test I, the shoulder motor torques required to perform the humeral flexion were computed (Figure 11(a)). It can be seen from the plot that the maximum torque is nearly 1.25 Nm. When the arm is crossing the vertical, the torques reach their minimum value. As the movement continues, the torques increase smoothly until they reached the maximum and then fall by the end of the motion. The angular displacement, the angular velocity, and the angular acceleration of the actuators were evaluated (Figures 11(b)–11(d)). It can be seen that the motors show a smooth movement. The range of the motion of the first and third motors is approximately 200°, and the second motor rotates 160°. There is an instant where motor 2 is stopped and then changes the direction of rotation. Along the entire movement, the angular acceleration is low until the last phase of the motion where motor 2 exhibits a significant acceleration. The mechanical power was calculated as the product of the torque and the angular velocity (Figure 11(e)). It can be seen that the maximum power is developed by motor 2 near the end of the motion with a value of 4 W. Regarding the elbow joint, despite no movement of this joint in this test, the torque required to maintain the elbow extended changes along the task ranging from 0 to almost 0.25 Nm (Figure 11(f)).

**Figure 12:**
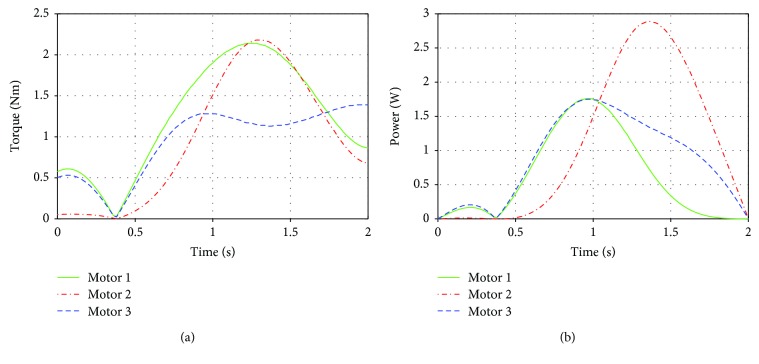
Results of Test I case b: (a) torques of the shoulder actuators and (b) power of the motors of the shoulder.

For the second case in Test I, when the parallel manipulator is actuated directly, the torque required is increased and the maximum torque is approximately 2.1 Nm, which is roughly 60% more than the highest torque of case a (Figure 12(a)). For this case, the maximum power is developed by motor 2 as in case a. The maximum value is 2.9 W (Figure 12(b)). This value is smaller than that in the first case; this could be explained due to the friction in the four-bar mechanism considered in case a.

**Figure 13:**
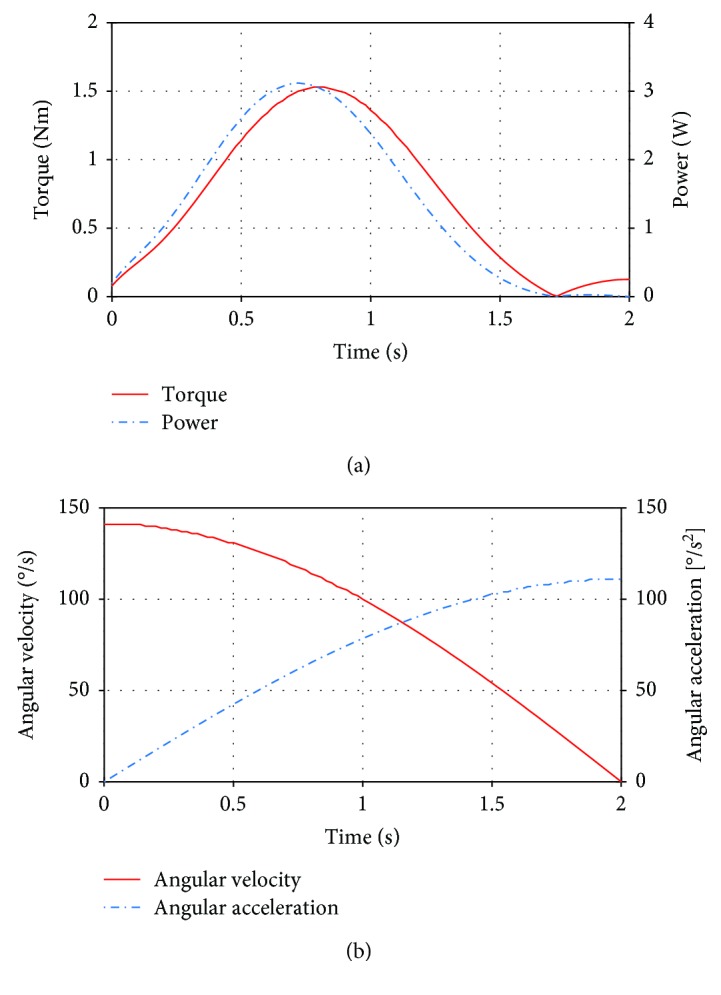
Results from Test II: (a) torque of the elbow actuator and mechanical power; (b) angular velocity and angular acceleration of the actuator.

In Test II, we evaluate the required torque for the elbow actuator to perform the flexion while the arm is in a vertical position. While the motion of the elbow continues, the torque increases until it reaches its maximum value of 1.5 Nm; then this value decreases as the four-bar mechanism of the elbow reaches its singular position (Figure 13(a), left *y*-axis). The power has a maximum value of 3.1 W (Figure 13(a), right *y*-axis). At the beginning and at the end of the movement, the power is low since the required torque is also low. The angular velocity (left *y*-axis) and angular acceleration (right *y*-axis) of the elbow actuator during this movement follow a sinusoidal wave (Figure 13(b)).

**Figure 14:**
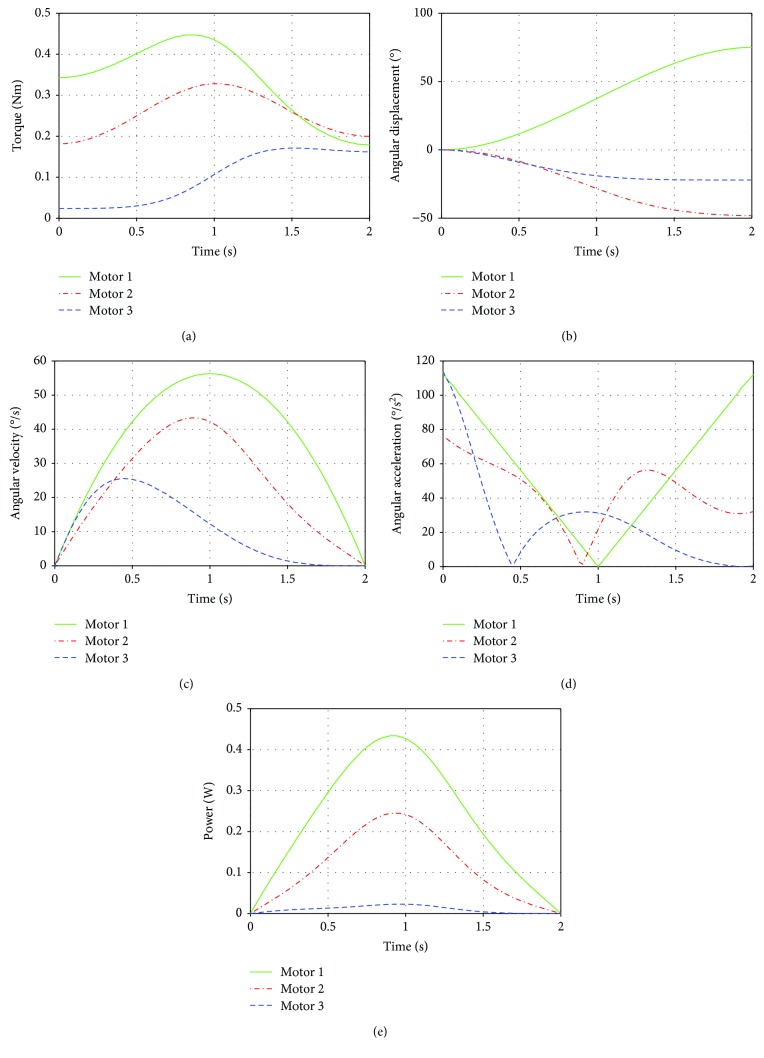
Results of torques of the wrist actuators during Test III: (a) wrist actuator torques; (b) angular displacement; (c) angular velocity; (d) angular acceleration; (e) mechanical power.

The functionality of the wrist is evaluated during Test III. In this test, as the load reaches the midpoint of the movement, where the lever arm of the wrist is maximum, the torques increase to their maximum value of 0.45 Nm; then it is reduced as the final position is reached (Figure 14(a)). The angular displacements, angular velocity, and angular acceleration of the motors present a smooth behavior, and the maximum range is approximately 70° (Figures 14(b)–14(d)). The power developed by the wrist motors during Test III has a maximum value of 0.42 W and is produced by motor 1 (Figure 14(e)). At the beginning and at the end of the movement, the power is close to 0.

## 8. Prototype Construction and Experimental Validation

A lab prototype of the prosthetic arm was built using a 3D printer Stratasys® Dimension 1200. The material used was ABS. For the parallel manipulator of the shoulder, three gear motors were used. This kind of motor works at 12 V, and the rated torque of the gearbox is 20 kg cm. At the joints of the shoulder mechanism, ball bearing 688zz was used (8 × 16 × 5 mm). The elbow was actuated with a servomotor model PDI-6221MG. This is a digital servomotor with a stall torque of 20 kg cm. Three MG996R servomotors were used to drive the wrist. These servomotors have a stall torque of 10 kg cm.  Table 1 summarizes the cost of the components of the prototype.

**Figure 15:**
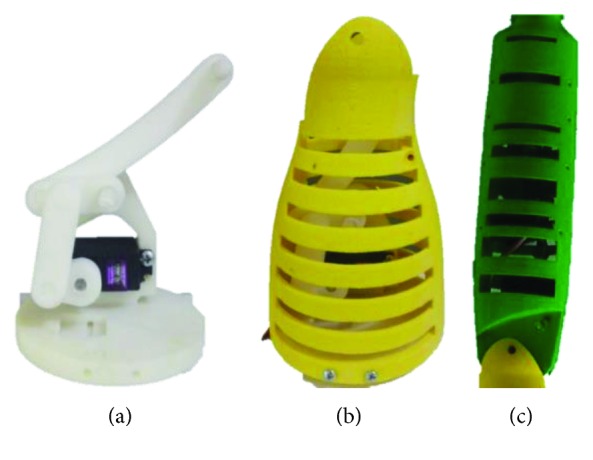
Different structures of the prototype: (a) actuator and the four-bar mechanism of the elbow, (b) arm structure, and (c) forearm structure.

The elbow motor was attached to the proximal part of the forearm (Figure 15(a)). Due to its reduced size, it can be easily fitted inside the arm, including the four-bar mechanism (Figure 15(b)). The wrist motors are fitted inside the forearm (Figure 15(c)) and actuate the wrist using bevel gears.

**Figure 16:**
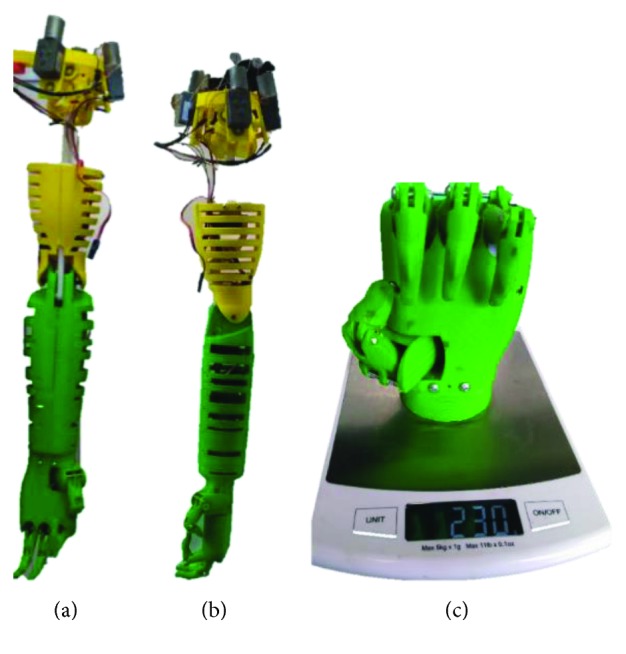
Prototype of the prosthetic arm: (a) frontal view; (b) lateral view; (c) hand attached to the prosthetic arm.

The previous elements and a prosthetic hand were assembled together to form the prosthetic arm (Figure 16).

The weight of the set arm-forearm-hand is 960 g, and the weight of the shoulder is 700 g. Therefore, the total weight of the device is 1660 g, which is lighter than that of a real arm. A comparison of the characteristics between the present design and the state-of-the-art solutions is found in Table 2.

Two movements of the prototype were experimentally assessed. The first movement was the flexion of the elbow, and the second movement was the humeral flexion.

To characterize the movements, the electric current of the motor using an ACS712 current sensor was measured. This sensor, based on a linear Hall sensor, is capable of measuring from 0 to 5 amperes. Its low offset makes it possible to use it without previous calibration and only using the gain of the output (185 mV/A). A GY-87 IMU sensor was used to measure the angular velocity and orientation of the forearm during the elbow flexion and the orientation of the arm during the humeral flexion. The IMU was attached to the forearm section of the prototype. The calibration of the gyroscope was performed measuring the reading offset in each axis while the IMU is not moving. The accelerometer was calibrated using the procedure that is reported in [17]. After calibration, the angular velocity is measured using the gain of the sensor and the orientation was calculated by performing a sensor fusion with a Kalman filter [18]. The data were acquired by using an Arduino Mega at a frequency of 50 Hz.

Considering that the servo tries to reach its final position as fast as possible, a subroutine was programed in order to send intermedium positions to the servomotor, and in this way, the speed is reduced to a proper level. This was performed to have a speed approximately equivalent to the 10% and 50% of the maximum operational speed (0.16 s/60°), namely, speed A and speed B, respectively, and speed C as the maximum speed.

## 9. Experiment Results

**Figure 17:**
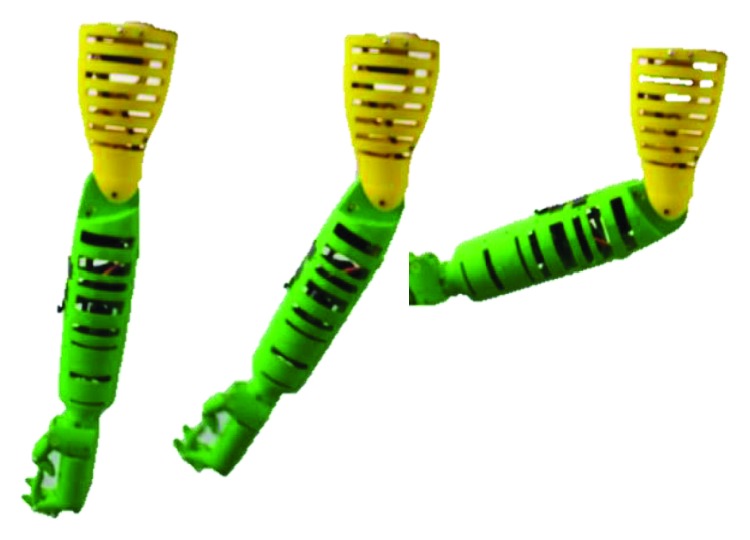
Snapchat of the elbow flexion experiment.

Elbow flexion was performed at three different speeds. The first experiment was at speed A, the second at speed B, and the third experiment at 100% of the speed of the motor. The motor was supplied with 6 V during the experiments. It can be seen that the forearm does not reach a horizontal position (Figure 17). This could be originated by the reduced stiffness of the links of the four-bar mechanism.

**Figure 18:**
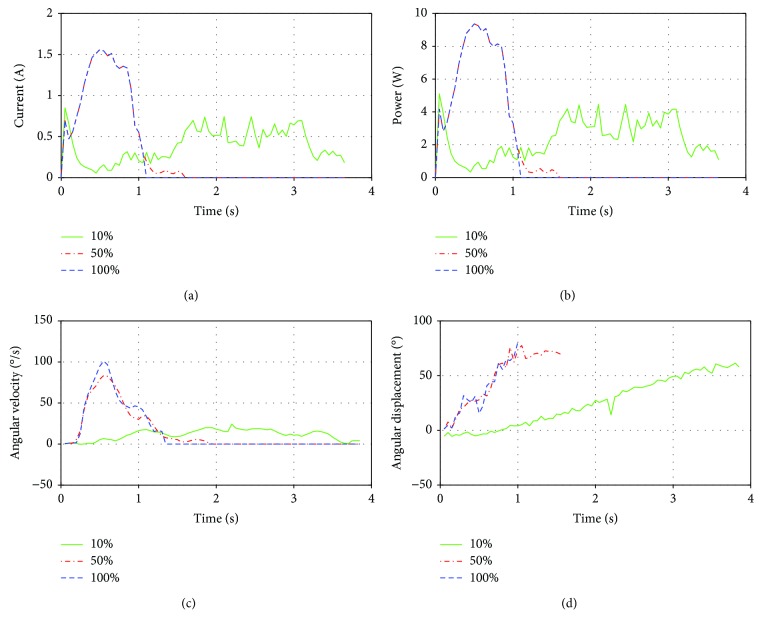
Results from experimental elbow flexion at speeds A, B, and C: (a) electrical current of the elbow motor; (b) electric power; (c) angular velocity; (d) angular displacement.

The results of the first experiment at speed A show that at the beginning of the movement, there is a peak of power consumption with a current of 2 A (Figure 18(a)). As the movement continues, the current is reduced to an average value of 0.7 A. The sudden decrease of the current could be originated as a result of the singular position of the mechanism at the beginning of the movement; then the current consumption rises as the lever arm increases while the movement continues. The lever arm reaches its maximum in the middle of the movement and then decreases as the current does. Considering that the voltage supplied was 6 V, the power consumption was calculated. The maximum angular velocity was 20°/s, and the duration of the movement was approximately 3.6 s that is too long to represent a natural movement of the elbow (Figure 18(b)). From Figure 18(c), it can be seen that the movement of the forearm follows a linear function. The range of the motion was approximately 65°; therefore, the entire elbow flexion is not completed.

When the speed of the experiment is set at speed B, the average current is increased to 1.5 A (Figure 18(a)). This represents almost 220% of the current at a velocity of 10%. It can be seen that after the initial peak, there is a drastic drop in the current, again because of the singularity of the mechanism. When the velocity is increased, there is an evident rise in the power consumption, showing a maximum consumption of 9 W. The maximum angular velocity developed during the movement is 85°/s, and it is reached at the midpoint of motion (Figure 18(b)). From Figure 18(d), it can be seen that the final position is reached at 1.6 s; this represents a reduction of the time in more than 100% compared with the previous experiment. This duration is acceptable for an elbow task.

When the speed is set at 100%, the average current is approximately 1.6 A (Figure 18(a)). Knowing that the voltage supply to the motor is 6 V, the power consumption, in this case, is around 9.5 W. In this case, the maximum angular velocity is 110°/s (Figure 18(b)). It can be seen that the duration is shorter than 1.1 s.

**Figure 19:**
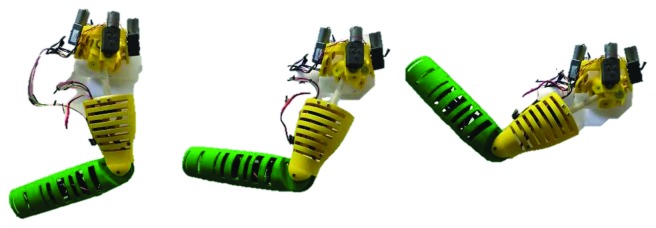
Sequence of the shoulder performing a humeral flexion movement.

After the evaluation of the elbow mechanism, the shoulder flexion with the prototype was performed (Figure 19). It can be seen that in this case, the arm does not reach a horizontal position but the range of motion is about 90°.

**Figure 20:**
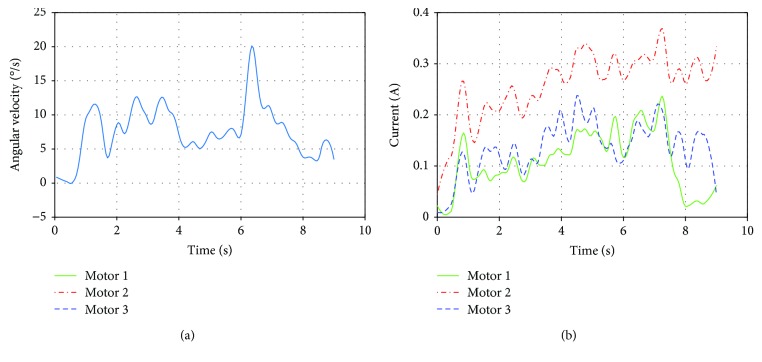
Results of the experimental shoulder flexion: (a) angular velocity of the arm and (b) electric current of the shoulder actuators.

During this experiment, the angular velocity was slow, having a mean value of 10°/s and the duration of the motion of 8.5 s (Figure 20(a)). It can be seen that at the beginning of the movement, there is a peak in the current that is higher in motor 2 (Figure 20(b)). The maximum average current required for this motion was 0.3 A.

The experimental validation shows that the proposed prototype is able to perform the movements with a reduced power consumption, but some elements of the device must be made of a different material in order to have enough strength to withstand the loads and not affect the operation of the mechanism.

## 10. Conclusions

In this work, a new human-like low-cost prosthetic arm has been designed. This device is formed by a three-DOF parallel manipulator at the shoulder, a six-bar mechanism of one DOF at the elbow, and a three-DOF spherical manipulator at the wrist, which are connected in a serial architecture. The spherical manipulator at the shoulder allows sharing the load, and therefore, the required torque and the power consumption of the motors are lower than those in other solutions. The use of small motors has the benefit that its weight is low and it is possible to create a design with a reduced cost and easier to afford than existing solutions. The shoulder mechanism makes it possible to place the actuator of the elbow close to the shoulder, and in this way, the inertial effects are reduced, in comparison to common solutions where the elbow is actuated with big motors placed at the elbow. The synthesis of this mechanism allows locking the elbow when it is fully extended or flexed; then the consumption of the elbow actuator can be reduced in these common positions. The wrist mechanism has a suitable range of motion to perform ADLs using small actuators. The selection of the mechanism makes it possible to have a prototype with a total weight of 1350 g, not including the hand. This weight is lower than the average weight of a human arm that is approximately 5 kg. The numerical and experimental evaluations show that the prototype can perform natural movements of the human arm.

Despite the feasibility of this device being demonstrated, further work needs to be done: this includes performing structural analysis in order to determinate the most suitable materials and dimensions of the main elements of the prosthesis aiming to assure its structural integrity, the implementation of compliant joints must be addressed to increase the safety for the user, and the establishment of an appropriate control scheme for this device is required.

## Figures and Tables

**Table 1:** Cost of the components used to build the prototype.

Component	Total price (USD)
3 gear motors	42
1 servomotor PDI-6221MG	13
3 servomotor MG966R	14
Bearings	6
3D-printed parts	215
Total	290

**Table 2:** Main characteristics of the proposed design and the state-of-the-art devices.

Author reference	DOF	Weight	Payload
[6]	7	4.8 kg	55 N
[7]	6	4.5 kg	—
[9]	7	4.45 kg	—
Present work	7	1.66 kg (included hand)	5 N

## Data Availability

The data used to support the findings of this study are available from the corresponding author upon request.
